# Genome-based reclassification of Kutzneria albida (Furumai et al. 1968) Stackebrandt et al. 1994 as a later heterotypic synonym of Kutzneria viridogrisea (Okuda et al. 1966) Stackebrandt et al. 1994

**DOI:** 10.1099/ijsem.0.007200

**Published:** 2026-06-16

**Authors:** Cecelia D. Dean, Richard W. McLaughlin, Mostafa S. Elshahed, Paul A. Lawson, Samuel L. Miller

**Affiliations:** 1 School of Liberal Arts and Sciences, Gateway Technical College, Kenosha, WI 53144, USA; 2 Department of Microbiology and Molecular Genetics, Oklahoma State University, Stillwater, OK 74074, USA; 3 School of Biological Sciences, University of Oklahoma, Norman, OK 73019, USA

**Keywords:** heterotypic synonym, *Kutzneria albida*, *Kutzneria viridogrisea*, *Kutzneria*

## Abstract

A comparison of 16S rRNA gene sequences between *Kutzneria albida* DSM 43870^T^ and *Kutzneria viridogrisea* DSM 43850^T^ revealed a sequence similarity of 99.8%. The genus *Kutzneria* includes five species with validly published names. All members of this genus have been isolated from soil samples collected in Japan or Thailand. The type strains *K. albida* DSM 43870^T^ (=ATCC 25243^T^=IFO 13901^T^=JCM 3240^T^=NBRC 13901^T^=NRRL B-24060^T^) and *K. viridogrisea* DSM 43850^T^ (=ATCC 25242^T^=IFO 15561^T^=JCM 3282^T^=NBRC 15561^T^=NRRL B-24059^T^=VKM Ac-1297^T^) were both isolated from soil samples from Japan. Whole-genome data were used to clarify the taxonomic assignment of these two closely related *Kutzneria* species. *K. albida* DSM 43870^T^ and *K. viridogrisea* DSM 43850^T^ share similar phenotypic and chemotaxonomic profiles. The major menaquinone and characteristic polar lipid are MK-9 (II, III-H_4_) and PII, respectively. Overall genomic relatedness analyses indicated that *K. albida* DSM 43870^T^ and *K. viridogrisea* DSM 43850^T^ shared average nucleotide identity and digital DNA–DNA hybridization values >95% and >70%, which are the currently accepted thresholds for species-level delineation. Based on the 16S rRNA gene phylogenetic tree and the core-genome phylogenomic trees, *K. albida* DSM 43870^T^ and *K. viridogrisea* DSM 43850^T^ phylogenetically form a separate, tight cluster apart from other *Kutzneria* species. Based on the combined evidence, *K. albida* (Furumai *et al*. 1968) Stackebrandt *et al*. 1994 is proposed to be a later heterotypic synonym of *K. viridogrisea* (Okuda *et al*. 1966) Stackebrandt *et al*. 1994.

The genus *Kutzneria* was named for the German microbiologist Hans-Jürgen Kutzner [1] and is a member of the family *Pseudonocardiaceae* within the phylum *Actinomycetota*. To date, *Kutzneria* includes five species with validly published names (https://lpsn.dsmz.de/genus/Kutzneria, February 2026). The type strains *Kutzneria albida* DSM 43870ᵀ and *Kutzneria viridogrisea* DSM 43850ᵀ were both isolated from soil samples collected in Japan [2,4]. *Kutzneria buriramensis* and *Kutzneria chonburiensis* were isolated from soil samples from Thailand [5,6]. Originally placed in the genus *Streptosporangium*, *K. viridogrisea*, *K. albida* and *K. kofuensis* were later transferred to the genus *Kutzneria* [1]. *K. albida* DSM 43870^T^ is Gram-stain-positive, non-acid-fast, aerobic, forms non-motile spores and aerial mycelia, and grows in up to 2.0% (w/v) NaCl [6]. Similarly, *K. viridogrisea* DSM 43850^T^ is Gram-stain-positive, non-acid-fast, aerobic, forms non-motile spores and aerial mycelia, and grows in up to 2.0% (w/v) NaCl [6]. Chemotaxonomically, *K. albida* DSM 43870^T^ and *K. viridogrisea* DSM 43850^T^ share a major quinone MK-9 (II, III-H_4_), phosphatidylethanolamine, hydroxyphosphatidylethanolamine, disphosphatidylglycerol and phosphatidylinositol as the major phospholipids (phospholipid type II); the cell wall contains *N*-acetylated muramic acid and *meso*-diaminopimelic acid [1]. Both strains also share iso-C_16 : 0_ and iso-C_16 : 0_ 2-OH [6] as their major fatty acids.

Comparison of the 16S rRNA gene sequences of *K. albida* DSM 43870ᵀ (EF543522) and *K. viridogrisea* DSM 43850ᵀ (MT760436) revealed 99.8% sequence similarity (Table 1), indicating an extremely close relationship, as this exceeds the 98.7% threshold value used in the assignment of strains to a species [7]. The EzBioCloud server [8] was subsequently used to confirm this relationship by retrieving 16S rRNA gene sequences from other members of the genus *Kutzneria* with validly published names. ClustalW was then used to align the sequences [9], and a phylogenetic analysis was constructed in mega12 [10], using the maximum-likelihood (ML) algorithm [11] (Fig. 1). Genetic distances were then calculated using the Kimura two-parameter model [12]. Finally, bootstrap values were calculated using the standard 1,000 replications [13]. The phylogenetic tree based on the 16S rRNA gene sequences further confirmed that *K. albida* DSM 43870^T^ and *K. viridogrisea* DSM 43850^T^ were closely related and formed a distinct clade separate from other *Kutzneria* species (Fig. 1).

**Fig. 1.**
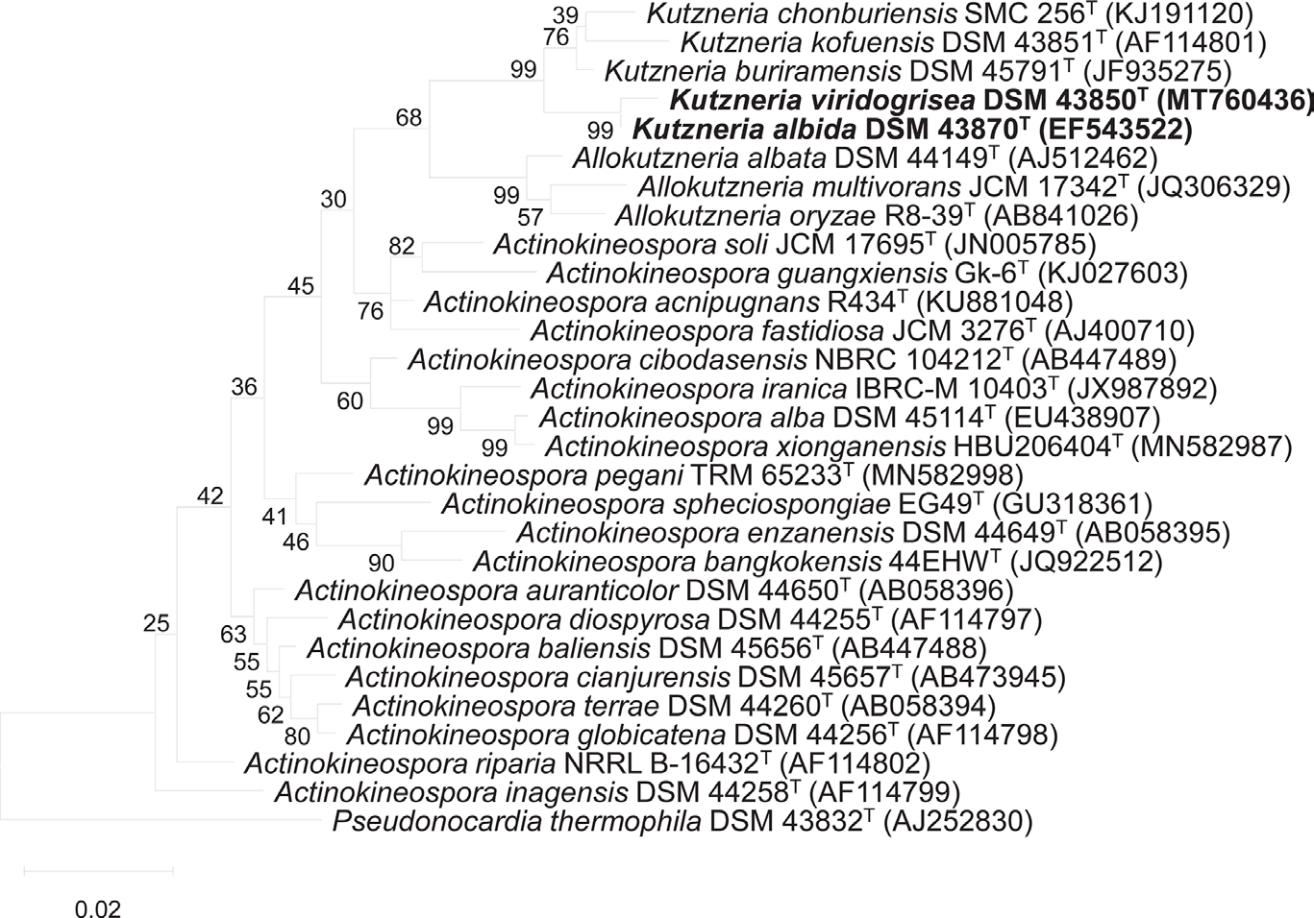
Phylogenetic tree based on 16S rRNA gene sequences (1,416 bp) of *K. albida* DSM 43870^T^ and *K. viridogrisea* DSM 43850^T^ and their nearest phylogenetic relatives. The tree was constructed using the ML method. Bootstrap values, expressed as percentages of 1,000 replications, are given at branching points. Database accession numbers are given in parentheses. Bar, 0.02 substitutions per nucleotide position.

**Table 1.** Pairwise comparison of 16S rRNA gene similarity and overall genomic relatedness indices between *K. albida* DSM 43870^T^ and *K. viridogrisea* DSM 43850^T^ and their close relatives

**16S rRNA gene similarity (%)**			
**Taxa**	**16S accession**	***K. albida***	***K. viridogrisea***
***K. albida***	**EF543522**	**100.0**	**99.8**
***K. viridogrisea***	**MT760436**	**99.8**	**100.0**
*K. buriramensis*	JF935275	98.3	98.0
*K. chonburiensis*	KJ191120	97.8	97.6
*K. kofuensis*	AF114801	97.6	97.5
**dDDH (%**)			
**Taxa**	**WGS accession**	***K. albida***	***K. viridogrisea***
***K. albida***	**GCA_000525635.1**	**100.0**	**94.4**
***K. viridogrisea***	**GCA_014138725.1**	**94.4**	**100.0**
*K. buriramensis*	GCA_003387475.1	21.7	21.7
*K. chonburiensis*	GCA_028622115.1	21.4	21.4
*K. kofuensis*	GCA_014203355.1	21.8	21.8
**ANI (%**)			
**Taxa**	**WGS accession**	***K. albida***	***K. viridogrisea***
***K. albida***	**GCA_000525635.1**	**100.0**	**99.4**
***K. viridogrisea***	**GCA_014138725.1**	**99.4**	**100.0**
*K. buriramensis*	GCA_003387475.1	80.6	80.6
*K. chonburiensis*	GCA_028622115.1	80.3	80.3
*K. kofuensis*	GCA_014203355.1	80.6	80.6

ANI, average nucleotide identity; dDDH, digital DNA–DNA hybridization; WGS, Whole Genome Sequence.

Genomic sequences are publicly available for *K. albida* DSM 43870^T^ and *K. viridogrisea* DSM 43850^T^ (GenBank accession numbers GCA_000525635.1 and GCA_014138725.1, respectively). To further explore the close phylogenetic relationship between *K. albida* DSM 43870^T^ and *K. viridogrisea* DSM 43850^T^, average nucleotide identity (ANI) [14] and digital DNA–DNA hybridization (dDDH) [15] values between the two strains and their close relatives were calculated. An orthoANI [16] value of 99.4% was observed between the two organisms, far exceeding the proposed species cut-off for ANI (95.0%–96.0%) [14]. This high level of relatedness was further confirmed using the Type (Strain) Genome Server [15], which showed that both genomes belonged to the same species, with a dDDH value of 94.4% (Table 1), which exceeds the accepted species cut-off boundary (>70.0%) [17]. A phylogenomic tree was reconstructed using the Codon Tree method [18] (Fig. 2). This method again demonstrated an extremely close relationship between *K. albida* DSM 43870^T^ and *K. viridogrisea* DSM 43850^T^ while showing separation from other *Kutzneria* species as in the 16S rRNA gene tree (Fig. 1).

**Fig. 2.**
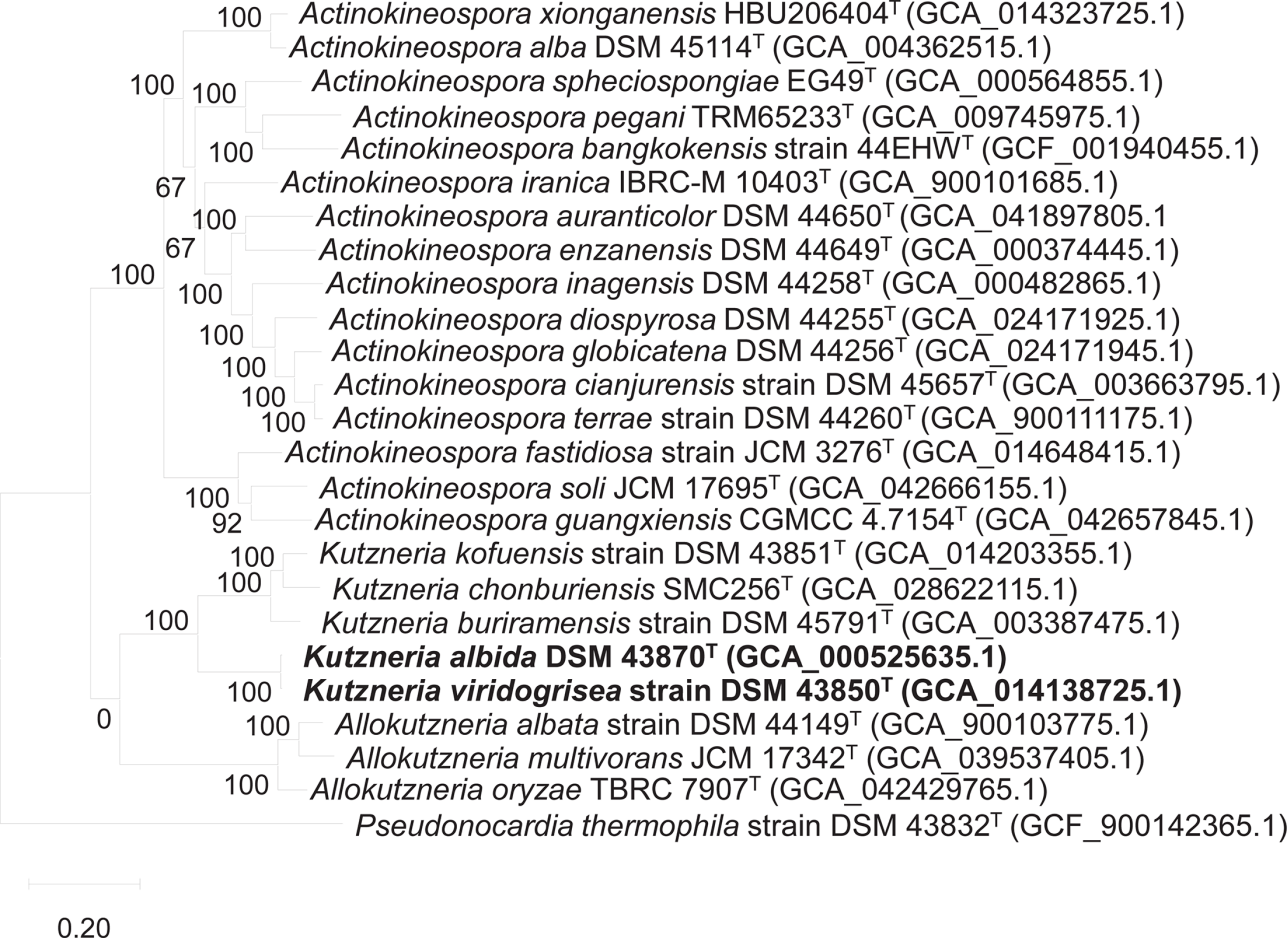
A core-genome phylogenomic tree based on an alignment of 100 concatenated single-copy genes showing the relationships among *K. albida* DSM 43870^T^ and *K. viridogrisea* DSM 43850^T^ and their nearest phylogenetic relatives. The tree was constructed using Codon Tree. Database accession numbers are given in parentheses. Bar indicates the mean number of substitutions per site, 0.2.

Comparison of the biochemical and physiological profiles of *K. albida* DSM 43870ᵀ and *K. viridogrisea* DSM 43850ᵀ revealed a high degree of similarity, including hypoxanthine hydrolysis and the utilization profiles of cellobiose, lactose, melibiose, d-ribose, d-xylose and l-rhamnose. The main phenotypic differences were the pigmentation of the mycelium and/or spores, as well as a limited number of phenotypic traits (Table 2). This is not surprising, given the high genetic similarity between the two strains (Table 1). These phenotypic differences are likely attributable to intraspecific variability and differences in assay sensitivity, which may account for the observed metabolic discrepancies.

**Table 2.** Differential physiological characteristics for *K. albida* DSM 43870^T^ and *K. viridogrisea* DSM 43850^T^ Data were taken from [6,20]; +, Positive; −, negative; w, weak.

Characteristic	*K. albida*	*K. viridogrisea*
Colour on ISP 2 Aerial mycelium or spore Substrate mycelium	Vivid greenish yellow Greenish white	Greenish white Greyish greenish yellow
Colour on ISP 3 Aerial mycelium or spore Substrate mycelium	Pale greenish yellow Deep yellow	Greenish white Greyish greenish yellow
Colour on ISP 5 Aerial mycelium or spore Substrate mycelium	Pale yellow Pale yellow	Greenish white Greenish white
Colour on JCM 42 Aerial mycelium or spore Substrate mycelium	Vivid greenish yellow Greenish white	Greenish white Pale greenish yellow
Sporangia size (µm)	11–30	21–50
Major menaquinone	MK-9(II, III-H_4_)	MK-9(II, III-H_4_)
Polar lipids	Type PII	Type PII
Hydrolysis of hypoxanthine	+	−
Whole-cell sugar	Rhamnose	Galactose and rhamnose
**Utilization of:**	–	–
Cellobiose	−	w
Lactose	−	+
Melibiose	w	+
d-Ribose	−	+
d-Xylose	w	+
l-Rhamnose	−	+

JCM 42 is yeast-starch agar.

ISP, International Streptomyces Project.

Collectively, we contend that, given the high relatedness based on overall genomic relatedness indices analyses (Table 1), the few physiological discrepancies reported (Table 2), and the high phylogenetic congruence observed in both the 16S rRNA gene and core-genome phylogenomic trees (Figs 1 and 2), *K. albida* DSM 43870^T^ and *K. viridogrisea* DSM 43850^T^ belong to the same species.

According to Rule ^24^b(4) of the International Code of Nomenclature of Prokaryotes [19], when two names compete for priority in the same publication, the author who first unites the taxa has the right to choose one of them. We therefore choose *Kutzneria viridogrisea* [4](Okuda et al. 1966) Stackebrandt *et al*. 1994 as the name for the united species, rendering *Kutzneria albida* [2](Furumai et al. 1968) Stackebrandt *et al*. 1994[1] its later heterotypic synonym. This choice is based on the priority of the original basonym *Streptosporangium viridogriseum* (1966) over *Streptosporangium albidum* (1968).

## Emended description of *Kutzneria viridogrisea*

The description is as before [1,6, 20] with the following modifications.

Grows at pH 5 and pH 9 and in the presence of 2% (w/v) NaCl. Reduces nitrate. Hydrolyses starch; hypoxanthine hydrolysis varies between strains. Utilises d-fructose, d-galactose, d-glucose, glycerol, d-mannitol, melibiose, raffinose and d-xylose as sole carbon sources, but not l-arabinose and salicin. Whole-cell sugars include galactose and rhamnose, although variation occurs among strains. The utilization of lactose, l-rhamnose and d-ribose as sole carbon sources varies between strains. When strain DSM 43870 was grown on International Streptomyces Project (ISP) 2 medium, the aerial mycelium or spores were vivid greenish yellow, whereas the substrate mycelium was greenish white. On ISP 3, the aerial mycelium or spores are pale greenish yellow, and the substrate mycelium is deep yellow. On ISP 5, the aerial mycelium or spores are pale yellow, and the substrate mycelium is pale yellow. On JCM 42 (yeast-starch agar), the aerial mycelium or spores are vivid greenish yellow, and the substrate mycelium is pale yellow. The sporangia are 11–50 µm in size. The genome size of the type strain is 10.2 Mb, and the DNA G+C content is 70.5 mol% (GCA_014138725.1).

The type strain is DSM 43850^T^ (=ATCC 25242^T^=IFO 15561^T^=JCM 3282^T^=NBRC 15561^T^=NRRL B-24059^T^=VKM Ac-1297^T^). Strain DSM 43870 (=ATCC 25243=IFO 13901=JCM 3240=NBRC 13901=NRRL B-24060) is an additional strain.

## Abbreviations

ANI: average nucleotide identity
dDDH: digital DNA–DNA hybridization
ISP: International *Streptomyces* Project
ML: maximum-likelihood
